# An RFID-Based Smart Nest Box: An Experimental Study of Laying Performance and Behavior of Individual Hens

**DOI:** 10.3390/s18030859

**Published:** 2018-03-14

**Authors:** Ying-Ren Chien, Yu-Xian Chen

**Affiliations:** Department of Electrical Engineering, National Ilan University, Yilan City 26047, Taiwan; R0441011@ms.niu.edu.tw

**Keywords:** Arduino, Internet of Things (IoT), smart agriculture, smart nest box, laying behavior, RFID

## Abstract

This study designed a radio-frequency identification (RFID)-based Internet of Things (IoT) platform to create the core of a smart nest box. At the sensing level, we have deployed RFID-based sensors and egg detection sensors. A low-frequency RFID reader is installed in the bottom of the nest box and a foot ring RFID tag is worn on the leg of individual hens. The RFID-based sensors detect when a hen enters or exits the nest box. The egg-detection sensors are implemented with a resistance strain gauge pressure sensor, which weights the egg in the egg-collection tube. Thus, the smart nest box makes it possible to analyze the laying performance and behavior of individual hens. An evaluative experiment was performed using an enriched cage, a smart nest box, web camera, and monitoring console. The hens were allowed 14 days to become accustomed to the experimental environment before monitoring began. The proposed IoT platform makes it possible to analyze the egg yield of individual hens in real time, thereby enabling the replacement of hens with egg yield below a pre-defined level in order to meet the overall target egg yield rate. The results of this experiment demonstrate the efficacy of the proposed RFID-based smart nest box in monitoring the egg yield and laying behavior of individual hens.

## 1. Introduction

Chicken eggs are of high nutritional value, providing amino acids, lipids, vitamins, and minerals. Egg farmers are primarily concerned with the yield and quality of eggs. The diet of laying hens can affect the nutritional quality of eggs [1], whereas the age of the hens and laying environment can affect egg yield [2]. Expected egg yield depends on the age and breed of the hens. Farmers generally monitor the egg yield of entire coops, such that a drop in egg yield prompts the replacement of all current hens with younger hens. This approach sacrifices hens that are still producing eggs at a satisfactory rate. Egg farms lack a systematic method to identify the production of individual hens. This study sought to employ the Internet of Things (IoT) and various automation technologies in the development of smart agriculture [3,4,5].

A review article [6] suggested that accelerometers, radio-frequency identification (RFID)-based sensors, and radio signal strength indicator (RSSI)-based sensors could be used to assess the activity and location of individual laying hens. The fact that the laying cycle of hens can exceed 50 weeks favors passive RFID-based sensors, which do not require an external power source. These devices can also be made small and light. RFID-based technology has been extensively adopted for human activities recognizing [7], human activities classification [8], object localization [9], path authentication [10], and farming [11].

Infrared technologies have been used in the development of a monitoring system for laying hens in a commercial organic egg farm [12,13]. However, the infrared technologies can only identify the presence of hens. The individual identity of hens is indistinguishable. Furthermore, a nest usage sensor based on the infrared sensor has been developed to detect the cases of double [14] or multiple [15] nest occupation. Photocells [15] or switches [16] have been used to detect individual eggs falling down an egg-collection tube. Thurner et al. [17] developed the funnel nest box (FNB) to track the individual laying productivity using low-frequency RFID technology (134.2 kHz).

With the FNB, Ichen et al. have conducted an individual laying performance test in noncage housing systems lasting over eight years [18]. There are about 3850 hens have been analyzed. The FNB-based data recording system was continuously ameliorated to improve its accuracy. A high accuracy of individually observed phenotypes, such as egg weight, oviposition time, and the duration of stay in a nest, etc., is helpful information to determine the genetic parameters. The inaccuracy mainly resulted from the double nest occupations and floor eggs. These problems can be significantly decreased by reducing the nest-to-hen ratio (approximately below 1:5.5 is a good choice). Thurner further developed a group nest box using a high-frequency RFID system to enable the simultaneous recording of individual laying behavior without the need to separate the hens [19,20].

Burel et al. [16] have proposed a plywood nest-box which contains four nests. However, this box needs double tags, one is injected in hen’s neck and the other is attached to its leg, to distinguish when a hen was entering the nest from when it was only looking into the nest. For laying performance and behavior of hens, most of automatically collect data systems identify hens through transponders (also called tags) fastened to legs or wings. In [21], the authors have evaluated the system performance with transponders injected into hens’ feet. Their experimental results have shown that the feet-injected transponders improve the hens’ identification speed.

In this paper, we developed an RFID-based IoT framework for smart agriculture applications, referred to as the smart nest box. We then conducted an experiment to analyze the laying performance and behavior of individual hens in enriched cages. The main contributions of this work are summarized as follows. First, we implemented an RFID-based IoT framework using Arduino as a micro-controller unit and WiFi shield as a daughter board to enable access to a cloud database and the network time protocol (NTP) server via the Internet. At the sensing level, each hen wore an RFID tag on one leg and an RFID reader was installed in each nest box to enable the detection of individual hens entering or leaving the nest box. Second, we also integrated a load cell, which is a resistance strain gauge pressure sensor, into the nest box as an egg-detection sensor. It can detect whether an egg is laid and its weight. Our egg-detection sensor is different from previous works, which used conventional switches [16], seesaw egg sensor [17] or photocells [15] to detect eggs. Furthermore, the egg weights are manually measured in the related works, but we can automatically weight the egg by using the same egg-detection sensor. Third, we designed a smart nest box integrating the proposed RFID-based IoT framework to enable the monitoring of laying performance and hen behavior in real-time. Fourth, we conducted an experiment to demonstrate the practicality of the scheme in an enriched cage. Based on the authors’ best knowledge, few works compared the individual yield rate with the group yield rate. Our proposed system enables egg farmers to use the egg yield of individual hens as a means of determining which hens should be culled.

## 2. Materials and Methods

### 2.1. Experimental Smart Nest Box

**Figure 1:**
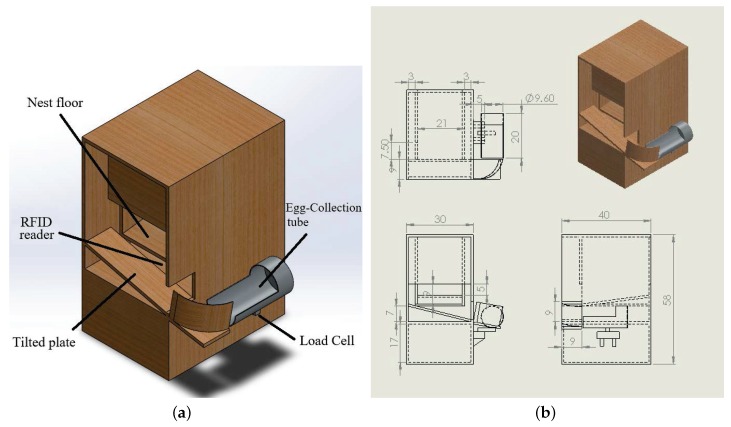
Design of Smart Nest Box (of 30 cm (W) × 40 cm (D) × 58 cm (H)). (**a**) Design drawing; (**b**) dimensional drawing.

Figure 1a illustrates the outward appearance of the proposed smart nest box. An RFID reader is located at the bottom of the box. Unlike the FNB proposed by Thurner, this device does not include a trap; however, it was designed to ensure that only one hen would be able to fit in the nest box to reduce the likelihood of double occupation [17]. Moreover, the nest floor where the hens stand is slightly tilted so that the possibility that two hens simultaneously stand on the nest floor is reduced. The dimensions of the nest box are detailed in Figure 1b. The floor of the nest has a specially designed slope that causes the egg to roll into an egg-collection tube behind the nest box via a tilted plate immediately after being laid. Beneath the egg-collection tube is installed a load cell, which serves as an egg-detection sensor as well as an egg-weight sensor. Our IoT platform queries the time server to obtain a timestamp whenever an egg is detected. All of the eggs laid in the smart nest box during one day accumulate in the egg-collection tube in the order that they were laid. Assigning each egg to an individual hen is achieved by combining the RFID tag identification (ID) on the hen with the timestamp and the position of the eggs in the egg-collection tube.

**Figure 2:**
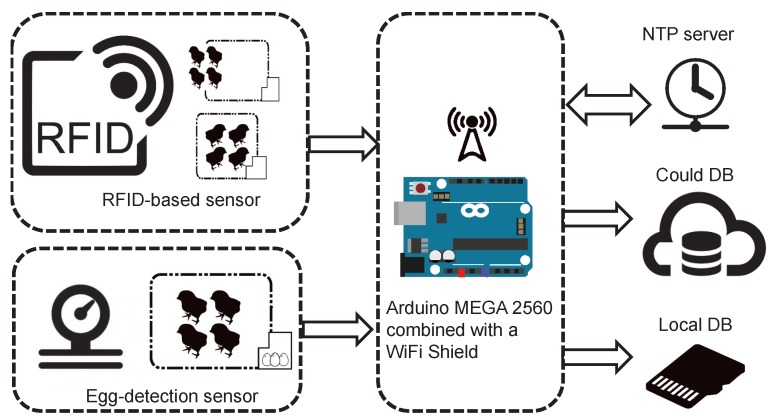
Block diagram of our IoT platform.

The core of the smart nest box is illustrated in Figure 2. At the sensing level, we have two types of sensors: a low-frequency RFID-based sensor to detect hens entering and exiting the smart nest box; and a resistance strain gauge pressure sensor for egg-detection.

**Figure 3:**
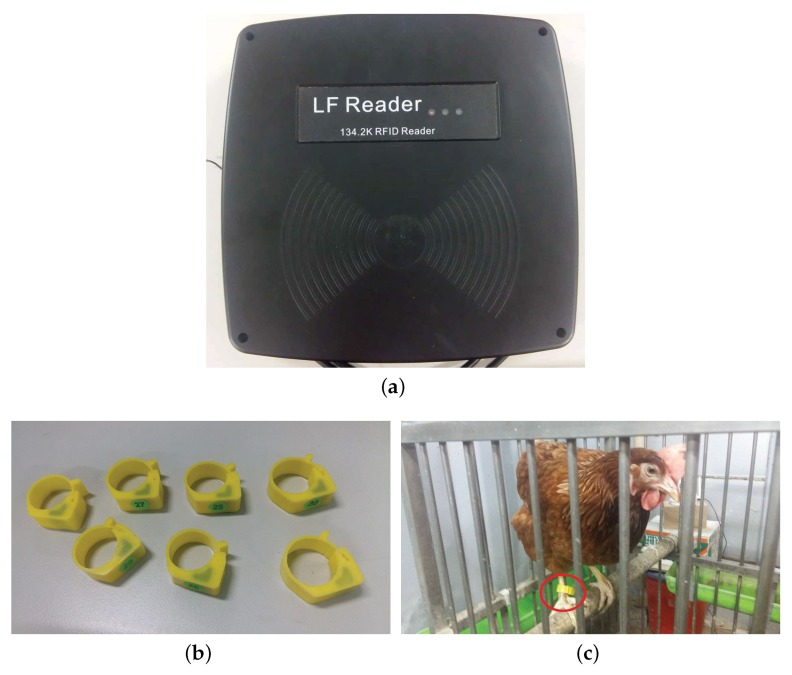
Photos of (**a**) an RFID reader, (**b**) foot ring RFID tags, and (**c**) a hen wears a foot-ring RFID tag.

The low-frequency RFID-based sensor system is comprised of an RFID reader (Figure 3a) and multiple passive RFID tags (Figure 3b). The size of reader antenna is $260\times 260\;{\mathrm{mm}}^{2}$ and the operation frequency is 134.2 kHz. The vertical and horizontal reading distances are 15 cm and 5 cm, respectively. Also, it is a full ISO11784/11785 compliant RFID reader with the RS-485 interface. Note that we need an extra interface module (MAX485) so that the IoT platform can correctly read the data stored in the tag. A photo of a hen that wears a foot ring RFID tag is shown in Figure 3c. The output of the pressure sensor is connected to a 24-bit analog-to-digital converter (ADC) (HX711). The resolution of this egg-detection sensor is 1 g and the maximum weight is 1 kg. The concept behind the egg detection scheme is simple. A new egg rolling into the egg-collection tube significantly alters the pressure in the tube (in the tens of grams).

The core of this IoT platform is an Arduino board (MEGA-2560), which was implemented using a daughter board (WiFi shield). The Arduino board queries the NTP server whenever a timestamp is required. The identity of a hen entering or exiting the nest box as well as the weight of the egg is recorded in a cloud database (DB). For debugging purposes, we also recorded these data on a local secure digital (SD) memory card as well.

**Figure 4:**
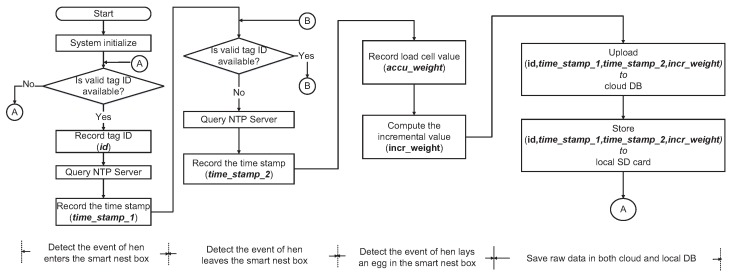
Flowchart of the IoT platform.

Figure 4 presents a flowchart of the proposed platform. During the initialization stage, the IoT platform validates the WiFi connectivity and sets all variables to their initial values. The platform then goes into monitoring mode to detect a hen entering the smart nest box. The serial port to which the RFID reader is connected is monitored until a valid tag ID (*id*) has been read. The platform then performs a query on a pre-defined NTP server to obtain a timestamp (*time_stamp_1*), denoting the time a hen entered the nest box. The program then goes into a status-checking loop used to detect a hen exiting the smart nest box. Our program keeps reading the data on the serial port until the valid tag ID becomes unavailable. The platform then performs a query on the NTP server to obtain another timestamp (*time_stamp_2*), denoting the time a hen exits the nest box. The program then reads the value output by the load cell, indicating the weight of all eggs in the egg-collection tube, which is an accumulated value (*accu_weight*). The weight of the newly laid egg is obtained by calculating the incremental value (*incr_weight*) via subtraction of the current *accu_weight (n)* from the previous *accu_weight (n-1)*, where *n* denotes the current time index. Note that the weight of an egg is normally in the tens of grams, which means that if *incr_weight* is less a given threshold (e.g., 10 g), then the system deems that no egg was laid between the time the hen with the ID (*id*) entered and exited the smart nest box. Otherwise, the hen with ID (*id*) is credited with laying an egg of *incr_weight* between *time_stamp_1* and *time_stamp_2*. Note that in this experiment, we did not record the time when an egg was exactly laid. The set of raw data *(id time_stamp_1, time_stamp_2, incr_weight)* is saved in the cloud database as well as a local SD card.

The cloud database was implemented by a MySQL server installed on a cloud-hosted virtual server. Our IoT platform can access the database by using PHP script through the HTTP protocol. The program returns to the detection phase to monitor hens entering the smart nest box.

### 2.2. Experimental Housing System

**Figure 5:**
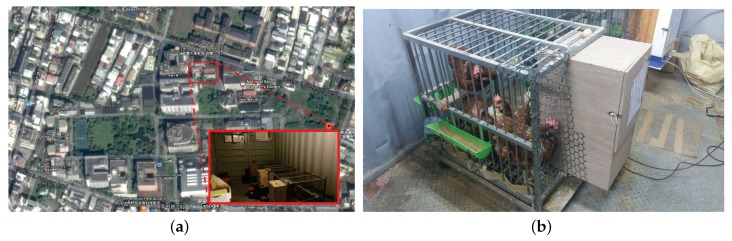
Experimental housing system. (**a**) Location of the experimental field; (**b**) an photo of the experimental housing system.

An experimental enriched cage housing system was used to evaluate the smart nest box. As shown in Figure 5a, the experimental housing system was located within a container house located on the campus of National Ilan University, Yilan city, Taiwan. As shown in Figure 5b, the enriched cage was equipped with three feeders, three water dispensers, one perch, and one smart nest box. The dimensions of the enriched cage were as follows: 80 cm (W) × 60 cm (D) × 70 cm (H). Four brown Lohmann hens were reared in the enriched cage. The perch was installed to enable the hens to perch at night.

### 2.3. Experiment Design

After arranging the coop environment and installing the relevant hardware and software, the experiment lasted a total of 45 days (from 30 April 2017 to 13 June 2017). The four hens reared in the enriched cage were given 14 d to adapt to the environment. A transponder (ISO 11784/85 compliant) was attached to the leg of each hen. The transponders tag IDs were as follows: 21, 22, 23, and 24. Data was collected from the smart nest box for the next 31 days (from 14 May 2017 to 13 June 2017). Throughout the experiment period, one person was given the task of visiting the coop every morning and evening to conduct the following tasks:

1. Clean the coop and ensure the temperature and humidity were within the required range.
2. Supply feed and water.
3. Turn the light in the coop in the morning (06:00–09:00) and off in the evening (16:30–18:00).
4. Collect the eggs in the egg-collection tube in the evening and record this number in laboratory record book.
5. Check the console of the IoT platform to ensure that no exceptions occurred.

**Figure 6:**
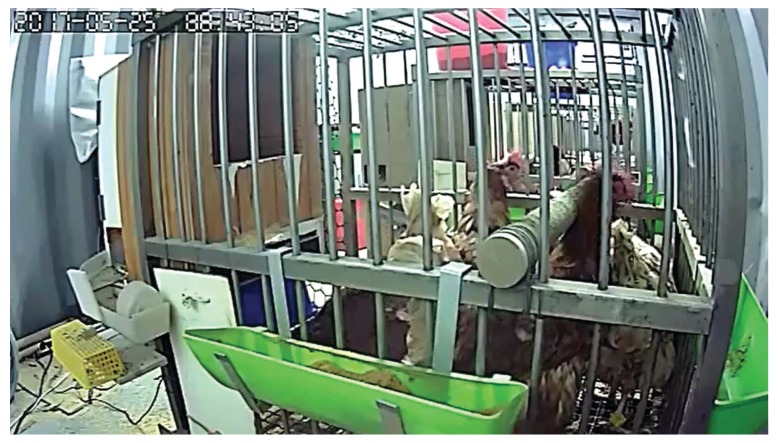
A snapshot from one recorded clip.

An infrared camera was installed to validate the accuracy of the RFID-based IoT platform. The angle and the focal length of the lens could be adjusted remotely. The video records captured by the infrared camera were used to compare with the events “when a hen enters the nest box”, “when a hen leaves the nest box”, and “when an egg was laid” that are detected by our RFID-based IoT platform. A snapshot from one recorded clip is presented in Figure 6.

## 3. Results and Discussion

### 3.1. Analysis of Egg Yield and Weight

Table 1 lists the daily egg production records for individual hen during the 31 days. “0” denotes that no egg was laid; “1” denotes that one egg was laid. Note that, although we have observed the double nest occupation, by carefully checking the recorded video, our daily egg production records are correct.

**Figure 7:**
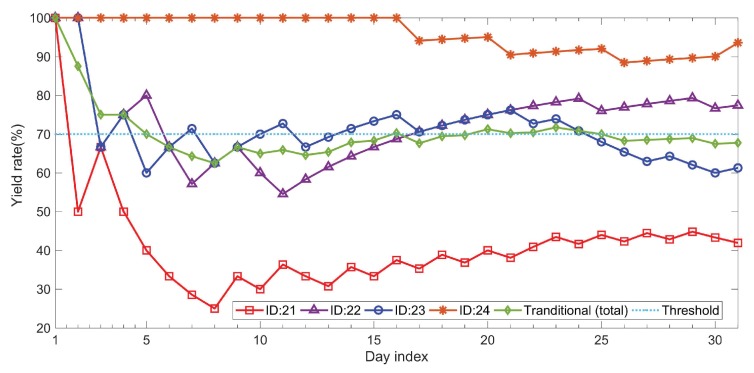
Individual vs. group yield rate.

**Figure 8:**
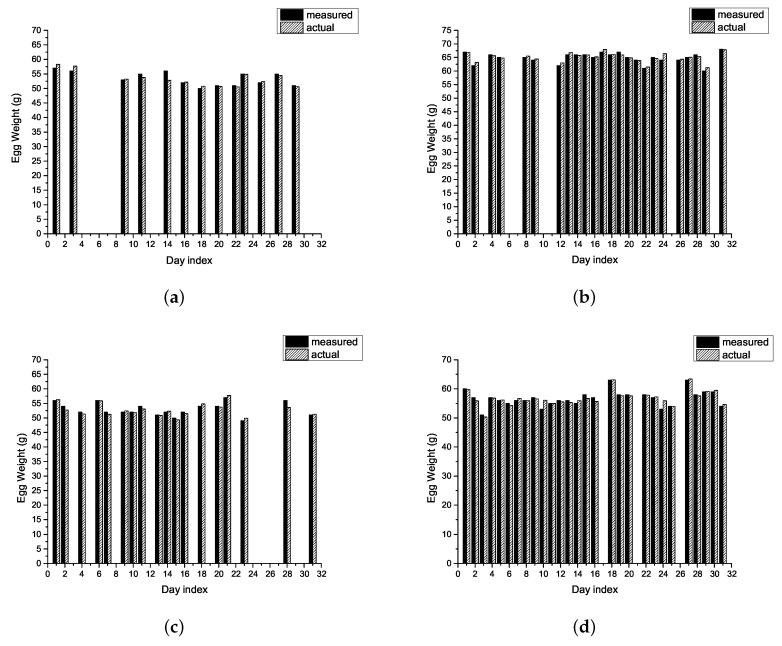
The daily measured vs. actual weights of eggs for the individual hens. (**a**) Hen with ID 21; (**b**) Hen with ID 22; (**c**) Hen with ID 23; (**d**) Hen with ID 24.

In this experiment, we kept four hens at 80 weeks. Thus, the group egg yield can be defined as follows:

(1) $$\begin{array}{c} r\left(\%\right)=\frac{m}{h\times d}\times 100\% \end{array}$$

where *m* is the total number of laid eggs within *d* days; h=4 is the number of hens in the group. Yield rate $r_{i}$ of the *i*-th hen is computed as follows:

(2) $$\begin{array}{c} r_{i}\left(\%\right)=\frac{m_{i}}{d}\times 100\% \end{array}$$

where $m_{i}$ is the number of eggs laid by the *i*-th hen within *d* days. Note that *h* equals 1 for the calculation of individual yield. Figure 7 presents the individual yield versus group yield. Most monitoring systems provide only the group egg yield; i.e., the yield of individual hens is disregarded. For an 80-week hen, the egg yield should be kept at 70%. However, without sophisticated techniques, such as RFID-based IoT solutions, egg farmers are unable to identify the egg-laying performance of individual hens or identify those that fail to meet expectations. Under these circumstances, the best policy dictates that a failure to meet yield expectations should lead to the replacement of the group with others younger hens. The proposed scheme was inspired by the idea of precise agriculture, wherein our RFID-based IoT system tracks the of individual hens, thereby enabling the identification of poorly performing hens. In this study, hens with the IDs 21 and 23 were underperforming. As shown in Figure 8a–d, the proposed scheme also provides the weights of eggs laid by individual hens. The values obtained from the load cell were compared with those obtained manually using an electronic precision scale (actual weights). The error between the measured and actual weights was less than ±0.7 g.

### 3.2. Nest Box Visiting and Laying Behavior

**Figure 9:**
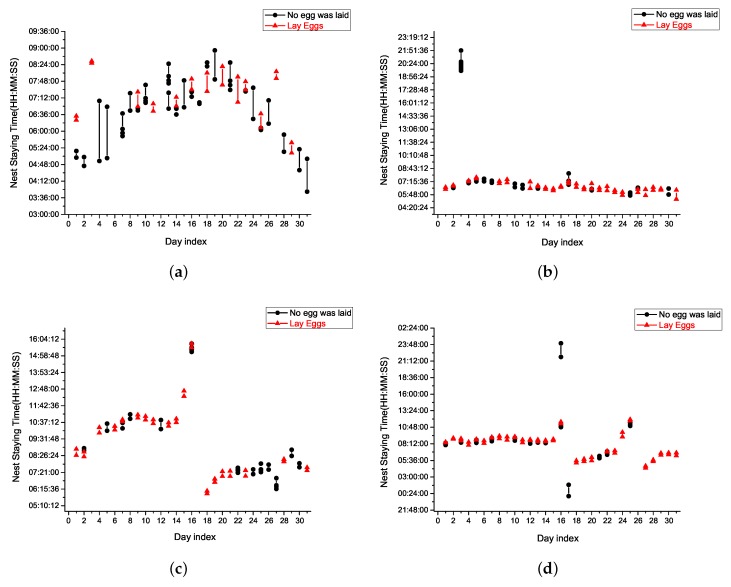
Individual hen laying behavior and the corresponding nest box visit duration. (**a**) Hen with ID 21; (**b**) Hen with ID 22; (**c**) Hen with ID 23; (**d**) Hen with ID 24.

The data recorded by the IoT platform can also be used to analyze the behavior of the hens with regard to visiting the nest box and laying eggs. Hens do not always lay eggs upon entering a nest box. Figure 9a–d illustrate the laying behavior of individual hens and the corresponding duration of visits to the nest box. In our experiment, these four hens exhibited different laying behavior. The nest staying durations for these four hens were different as well. There were only some hours of a day during which the hens visited the nest, as shown in Figure 9. Note that although the space in the nest box was sufficient to accommodate only one hen, this did not eliminate cases of double occupation. On the first and ninth days, hens with IDs 21 and 22 occupied the nest simultaneously, whereas on the twenty-ninth day, hens with IDs 22 and 24 occupied the nest simultaneously. The total number of nest box visiting was 174 and the number of double nest occupations was 3 times. Thus, the double nest occupation ratio was approximately 1.72%. Table 2 summarizes Figure 9a–d, where $N_{1}$ denotes the number of times the nest box was visited without an egg being laid, $N_{2}$ denotes the number of times the nest box was visited and an egg was laid by a particular hen. The ratio of $N_{2}/\left(N_{1}+N_{2}\right)\times 100\%$ represents the percentage of nest box visits in which an egg was laid by a particular hen. These results coincide with the yield of individual hens. In this situation, the hen that should be replaced is ID 21. This kind of information is helpful for egg farmers.

**Figure 10:**
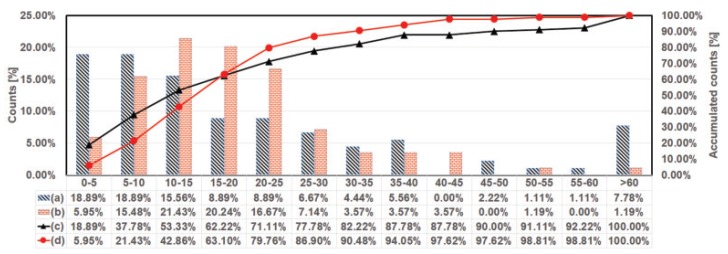
Histogram of visiting duration for all hens. (**a**) Without lay eggs; (**b**) With lay eggs; (**c**) Accumulated amount of hens without nest visits with lay eggs; (**d**) Accumulated amount of hens with nest visits with lay eggs.

Figure 10 presents histograms of visit-duration for all of the hens. For more than 50% of the hens that visited the nest box without lay eggs, their visiting durations were less than 15 min. For more than 90% of the hens that visited the nest box with lay eggs, their visiting durations were less than 35 min.

## 4. Conclusions

This paper presents the design of a smart nest box based on the RFID-based IoT platform. This IoT platform connects the hens and laid eggs with the Internet. The smart nest box makes it possible to detect egg-laying events, analyze the egg yield of individual hens, measure egg weights, and analyze nest box visiting behavior. By focusing on individual yields, the proposed system is able to identify hens that fail to meet yield expectations, thereby making the replacement of underperforming hens more efficient. Via accessing the cloud DB, egg farmers know the egg yield of individual hens and which hens are not laying in real time. Such hens can be culled to avoid wasting feeds. In addition, farmers know the number of eggs produced per day and their weights. This might prevent workers from stealing the laid eggs.

## Figures and Tables

**Table 1:** Daily egg production records for the individual hens.

**Day Index**	**1**	**2**	**3**	**4**	**5**	**6**	**7**	**8**	**9**	**10**	**11**	**12**	**13**	**14**	**15**	
ID: 21	1	0	1	0	0	0	0	0	1	0	1	0	0	1	0	
ID: 22	1	1	0	1	1	0	0	1	1	0	0	1	1	1	1	
ID: 23	1	1	0	1	0	1	1	0	1	1	1	0	1	1	1	
ID: 24	1	1	1	1	1	1	1	1	1	1	1	1	1	1	1	
Total	4	3	2	3	2	2	2	2	4	2	3	2	3	4	3	
**Day Index**	**16**	**17**	**18**	**19**	**20**	**21**	**22**	**23**	**24**	**25**	**26**	**27**	**28**	**29**	**30**	**31**
ID: 21	1	0	1	0	1	0	1	1	0	1	0	1	0	1	0	0
ID: 22	1	1	1	1	1	1	1	1	1	0	1	1	1	1	0	1
ID: 23	1	0	1	1	1	1	0	1	0	0	0	0	1	0	0	1
ID: 24	1	0	1	1	1	0	1	1	1	1	0	1	1	1	1	1
Total	4	1	4	3	4	2	3	4	2	2	1	3	3	3	1	3

**Table 2:** Number of visiting times of nest box for the individual hens.

Hen ID	$N_{1}$	$N_{2}$	$N_{2}/\left(N_{1}+N_{2}\right)$ (%)
21	27	13	32.50%
22	25	24	48.98%
23	20	19	48.72%
24	18	28	60.87%

## References

[1] Gładkowski W., Kiełbowicz G., Chojnacka A., Gil M., Trziszka T., Dobrzański Z., Wawrzeńczyk C. (2011). Fatty acid composition of egg yolk phospholipid fractions following feed supplementation of Lohmann Brown hens with humic-fat preparations. Food Chem..

[2] Xin H., Gates R.S., Green A.R., Mitloehner F.M., Moore P.A., Wathes C.M. (2011). Environmental impacts and sustainability of egg production systems. Poult. Sci..

[3] Ruiz-Garcia L., Lunadei L., Barreiro P., Robla I. (2009). A Review of Wireless Sensor Technologies and Applications in Agriculture and Food Industry: State of the Art and Current Trends. Sensors.

[4] Chen H., Xin H., Teng G., Meng C., Du X., Mao T., Wang C. (2016). Cloud-based data management system for automatic real-time data acquisition from large-scale laying-hen farms. Int. J. Agric. Biol. Eng..

[5] Popović T., Latinović N., Pešić A., Zečević Ž., Krstajić B., Djukanović S. (2017). Architecting an IoT-enabled platform for precision agriculture and ecological monitoring: A case study. Comput. Electron. Agric..

[6] Siegford J.M., Berezowski J., Biswas S.K., Daigle C., Gebhardt-Henrich S., Hernandez C., Thurner S., Toscano M. (2016). Assessing Activity and Location of Individual Laying Hens in Large Groups Using Modern Technology. Animals.

[7] Yao L., Sheng Q.Z., Li X., Gu T., Tan M., Wang X., Wang S., Ruan W. (2018). Compressive Representation for Device-Free Activity Recognition with Passive RFID Signal Strength. IEEE Trans. Mob. Comput..

[8] Cornacchia M., Ozcan K., Zheng Y., Velipasalar S. (2017). A Survey on Activity Detection and Classification Using Wearable Sensors. IEEE Sens. J..

[9] Subedi S., Pauls E., Zhang Y.D. (2017). Accurate Localization and Tracking of a Passive RFID Reader Based on RSSI Measurements. IEEE J. Radio Freq. Identif..

[10] Bu K., Li Y. (2018). Every Step You Take, I Will Be Watching You: Practical StepAuth-Entication of RFID Paths. IEEE Trans. Inf. Forensics Secur..

[11] Taylor K., Griffith C., Lefort L., Gaire R., Compton M., Wark T., Lamb D., Falzon G., Trotter M. (2013). Farming the Web of Things. IEEE Intell. Syst..

[12] Zaninelli M., Redaelli V., Tirloni E., Bernardi C., Dell’Orto V., Savoini G. (2016). First Results of a Detection Sensor for the Monitoring of Laying Hens Reared in a Commercial Organic Egg Production Farm Based on the Use of Infrared Technology. Sensors.

[13] Zaninelli M., Redaelli V., Luzi F., Bontempo V., Dell’Orto V., Savoini G. (2017). A Monitoring System for Laying Hens That Uses a Detection Sensor Based on Infrared Technology and Image Pattern Recognition. Sensors.

[14] Zaninelli M., Costa A., Tangorra F., Rossi L., Agazzi A., Savoini G. (2015). Preliminary Evaluation of a Nest Usage Sensor to Detect Double Nest Occupations of Laying Hens. Sensors.

[15] Zaninelli M., Redaelli V., Luzi F., Mitchell M., Bontempo V., Cattaneo D., Dell’Orto V., Savoini G. (2018). Development of a Machine Vision Method for the Monitoring of Laying Hens and Detection of Multiple Nest Occupations. Sensors.

[16] Burel C., Ciszuk P., Wiklund B.S., Brännäs E., Kiessling A. (2002). Note on a method for individual recording of laying performance in groups of hens. Appl. Anim. Behav. Sci..

[17] Thurner S., Wendl G., Preisinger R. Funnel nest box: A system for automatic recording of individual performance and behaviour of laying hens in floor management. Proceedings of the XII European Poultry Conference.

[18] Icken W., Thurner S., Heinrich A., Kaiser A., Cavero D., Wendl G., Fries R., Schmutz M., Preisinger R. (2013). Higher precision level at individual laying performance tests in noncage housing systems. Poult. Sci..

[19] Simultaneous Registration of Hens in Group Nest Boxes with a HF-Transponder-System to Evaluate the Laying Behavior. https://geoscience.net/research/033/398/033398053.php.

[20] Reiners K., Hegger A., Hessel E.F., Böck S., Wendl G., den Weghe H.F.V. (2009). Application of RFID technology using passive HF transponders for the individual identification of weaned piglets at the feed trough. Comput. Electron. Agric..

[21] Zaninelli M., Rossi L., Costa A., Tangorra F.M., Guarino M., Savoini G. (2016). Performance of injected RFID transponders to collect data about laying performance and behaviour of hens. Large Anim. Rev..

